# The relationship between different C-peptide level and insulin dose of insulin pump

**DOI:** 10.1038/s41387-020-00148-7

**Published:** 2021-01-22

**Authors:** Yihan Wei, Li Quan, Ting Zhou, Guoli Du, Sheng Jiang

**Affiliations:** State Key Laboratory of Pathogenesis, Prevention and Treatment of High Incidence Diseases in Central Asia; Department of Endocrinology, The First Affiliated Hospital of Xinjiang Medical University, Urumqi, 830017 China

**Keywords:** Type 2 diabetes, Carbohydrates

## Abstract

**Background:**

This study aims to explore the insulin requirement profiles, and analyze the related factors of type-2 diabetes mellitus (T2DM) with different C-peptide levels on insulin pump therapy.

**Methods:**

A retrospective study was conducted on 271 T2DM patients treated with insulin pumps from 2016 to 2018. These patients were divided into groups according to the ratio of C-peptide at 2 h after meals to fasting C-peptide (C_2_h/C_0_), and the dosage of insulin and influencing factors were analyzed.

**Results:**

In comparing group A (C_2h_/C_0_ < 2.5) with group B (C_2h_/C_0_ ≥ 2.5), the percentage of the base amount in total (%TBa, 0.50 ± 0.06) in group A was higher than that in group B (0.48 ± 0.05) (*P* < 0.05). Furthermore, there was a correlation between C_2h_/C_0_ and waist circumference, HbA1c, Fasting Plasma Glucose (FPG) and Blood glucose 2 h after meal (2hPG) (*r* = −0.137, −0.154, −0.471, and −0.172; all, *P* < 0.05). The multiple linear regression analysis revealed that BMI and FPG were independent factors of %TBa (β′ = 0.124 and 0.144; all, *P* < 0.05), and BMI and FPG were independent factors of C_2h_/C_0_ (β′ = −0.134 and −0.502; all, *P* < 0.05).

**Conclusions:**

The basal premeal dose ratio of T2DM with different C-peptide levels differs during intensive insulin pump therapy. Parameters that indicate the glycemic control and β-cell function should be taken into consideration for total insulin requirements.

## Introduction

Type-2 diabetes mellitus (T2DM) is one of the most frequent metabolic diseases with high incidence^1^. At present, the incidence of T2DM in adults has reached 10.4% in China^1^. Therefore, early diagnosis and early treatment are particularly important for patients with T2DM^2^. Early intensive treatment can quickly control blood glucose levels, reduce the severity of glucotoxicity, and recover the islet function in varying degrees^2,3^. Thus, due to precise and flexible dosage adjustment, the insulin pump can quickly control the blood glucose, reduce insulin dosage, and reduce the occurrence of hypoglycemia, which is an important means of intensive insulin therapy^4^. The impact of the diet on glycemic control but also on complications is really important^5^.

C-peptide is secreted by islet β cells, and has a common precursor proinsulin with insulin. Therefore, proinsulin can be decomposed into one molecule of insulin and one molecule of C-peptide. Thus, the molar weight of C-peptide is the same as that of a patient’s self-secreted insulin^5^. Furthermore, it is not easy for C-peptide to be degraded by the liver. Hence, this can reflect the content of insulin in the body through the detection of the level of C-peptide, and accurately reflect the function of islet cells^6^. Since the level of C-peptide is not influenced by exogenous insulin, the serum C-peptide level at 2 h after the fasting blood glucose loading test can be measured at the same time with the oral glucose tolerance test. The function of islet β-cells may be reflected by the ratio of the C-peptide level at 2 h postprandial (C_2h_) to the fasting C-peptide level (C_0_) (C_2h_/C_0_)^7^. However, at present, few studies have been conducted on the initial dosage setting of an insulin pump in T2DM, and the existing method mainly refers to the normal human insulin secretion mode and the application experience of patients with type-1 diabetes mellitus (T1DM)^8^. Hence, there is still a lack of studies on the design of insulin dosage based on C2h/C0^8^.

The present study aimed to summarize the characteristics of the insulin dosage ratio in patients with T2DM with different C-peptide levels when the blood glucose level reaches the target, and analyzed the relevant influencing factors, in order to provide a clinical basis for the initial dosage setting of insulin pumps for patients with T2DM.

## Research methods

### Objects

From December 2016 to December 2018, 271, inpatients with T2DM in the Department of Endocrinology, the First Affiliated Hospital of Xinjiang Medical University were enrolled in the present retrospective study. The inclusion criteria were as follows: the diagnosis was in accordance with the World Health Organization (WHO) diagnostic criteria in 1999; patients with T2DM who were in need of short-term or long-term intensive insulin therapy; patients without severe acute complications; patients without severe cardiac, hepatic and renal dysfunction. The exclusion criteria were as follows: patients with T1DM, and patients who use insulin secretagogue. These patients were divided into two groups: group A with C2h/C0 < 2.5 ng/ml and group B with C2h/C0 ≥ 2.5 ng/ml. The islet cell antibody (ICA), glutamic acid decarboxylase antibody (GAD-Ab), and insulin autoantibody (IAA) were all negative in these patients. Patients received diabetes education since admission, and adhered to a diabetic diet (containing 50–55% carbohydrates, 25–30% fat, and 15–20% protein, total calories (25–30)kcal·kg(ideal weight)· D-1, distributed to three meals according to 1:2:2).

The study was conducted in accordance with the Declaration of Helsinki (as was revised in 2013). The study was approved by Ethics Committee of The First Affiliated Hospital of Xinjiang Medical University and informed consent was taken from all the patients.

### Methods

The clinical indexes related to diabetes and the condition concerning the use of insulin pumps were recorded according to medical records. The patients were under fasting conditions for 10 h, and the body height, body weight, waist circumference, and BMI were measured the next morning after hospitalization. ICA, GAD, IAA, and HbA1c were detected in the blood samples drawn from these patients. Oral glucose tolerance test (OGTT) (75 g of anhydrous glucose or 100 g of steamed bread meal) was used to detect the fasting plasma glucose (FPG), fasting C-peptide (FC-P), plasma glucose 2-h postprandial (2hPG) and C-peptide 2-h postprandial (2hC-P), and 2-h postprandial, respectively. The insulin pump therapy was subsequently performed. Insulin analogues (insulin aspart or insulin lysine) were used as the pump insulin. According to present guidelines for the use of insulin pumps, the initial insulin dosage was 0.5 u·kg^−1^·d^−1^, and the basal dosage (TBa) was given at a proportion of 1:1 with the premeal dosage. During the period of insulin pump therapy, the basal rate and premeal dosage were adjusted according to the fingertip blood glucose level (detected using a Roche blood glucose meter (Accu-Chek Inform II) until the fingertip blood glucose reached the targets (Fasting Plasma Glucose (FPG) ≤ 7.0 mmol/l, Blood glucose 2 h after meal (2hPG) ≤ 10.0 mmol/l). The average duration of insulin pump use was (7.31 ± 2.56) days. Hypoglycemia was defined as having hypoglycemic symptoms and/or the level of peripheral blood glucose was <3.9 mmol/l. The usage of insulin was recorded for three days after the blood glucose reached the targets. C-peptide was detected by radioimmunoassay, FPG and 2hPG was detected using the glucose oxidase method, HbA1c was analyzed by high-pressure liquid chromatography, and the lipid profiles were detected using the enzymatic method. This study included in cases of patients with type-2 diabetes, improve diabetes antibody five determination after admission, including insulin resistance cell antibody (40 kd), islet cell antibodies (60 kd) resistance, resistance to IA - 2 a islet cell antibody (anti tyrosine antibody), resistance to insulin antibodies (IAA), resistance to glutamic acid decarboxylase antibody results were negative. The reference interval of this test in our hospital was negative, and there was no defined range.

### Statistical analysis

The SPSS 22.0 software was used for the statistical analysis. Measurement data were expressed as mean ± standard deviation. All measurement data were normally distributed as tested by Shapiro–Wilktest. Two-independent samples *t*-test was used for group comparisons. Pearson correlation analysis was used for the correlation analysis, and linear regression analysis was used for the multivariate analysis. *P* < 0.05 was considered statistically significant.

## Results

### General data

A total of 271 patients with T2DM were enrolled in the present study. Among these patients, 195 were males (72%) and 76 were females (28%). As presented in Table 1, the average age of these patients was 52.9 ± 11.9 years old, and the mean of the duration of T2DM was [5 (1, 12)] years. The mean BMI was 25.30 ± 3.47 kg/m^2^, and the mean FPG and 2hPG was 10.51 ± 3.36 mmol/L and 20.52 ± 4.27 mmol/L, respectively. Furthermore, the mean FC-P and 2hC-P was 1.25 ± 0.70 ng/ml and 2.65 ± 1.41 ng/ml, respectively. The other clinical characteristics are presented in Table 1. During the period of insulin pump therapy, the average incidence of hypoglycemia was 0.04 ± 0.19 times/case. Furthermore, after 3 days of insulin using, 96.3% of patients did not have hypoglycemia, while 3.4% of patients had 1–3 times of hypoglycemia, 3% had more than 3 times of hypoglycemia. The average time for reaching the therapeutic targets was 3.94 ± 1.02 days (Table 1). Fasting blood glucose was (10.51 ± 3.36) mmol/L, and blood glucose was (20.52 ± 4.27) mmol/L for 120 min.

**Table 1:** Clinical characteristics of T2DM receiving intensive insulin pump therapy.

Clinical features	Results ($\overline x \pm s$)
Age (years)	52.9 ± 11.9
Duration of disease (year)	7.11 ± 6.78
Weight (Kg)	72.56 ± 12.07
Waist circumference (cm)	94.58 ± 11.53
Body mass index (kg/m^2^)	25.30 ± 3.47
HbAlc (%)	11.1 ± 1.8
Fasting blood glucose (mmol/L)	10.51 ± 3.36
Blood glucose at 2-h after meal (mmol/L)	20.52 ± 4.27
Fasting plasma C-peptide (ng/ml)	1.25 ± 0.70
Postprandial 2hc peptide (ng/ml)	2.65 ± 1.41
TG (mmol/L)	2.59 ± 2.51
TC (mmol/L)	4.51 ± 1.15
HDL-C (mmol/L)	1.06 ± 0.13
LDL-C (mmol/L)	2.98 ± 0.93
Average occurrence times of hypoglycemia (times/cases)	0.04 ± 0.19
Mean blood glucose reaching standard time (day)	3.94 ± 1.02

### Grouping analysis

#### Grouping according to the median of C_2h_/C_0_ (2.5 ng/ml)

According to the median value of C2h/C0 (2.5 ng/ml) before insulin pump therapy, the patients were divided into two groups^9^: group A, C_2h_/C_0_ < 2.5 ng/ml, *n* = 168 cases; group B, C_2h_/C_0_ ≥ 2.5 ng/ml, *n* = 103 cases. The FPG, 2hPG, BMI, HbAlc, and insulin dosages were compared between the two groups. As presented in Table 2, the levels of FPG, 2hPG, BMI, and HbAlc were higher in group A, when compared to group B, and the difference was statistically significant (*P* < 0.05). The mean basal rate within 24 h (TBa/h) and the percentage of basal insulin in the total (%TBa) were higher in group A than in group B, and the difference was statistically significant (*P* < 0.05). However, there was no statistical significance in the difference in dosage of insulin per body weight (TDD/kg) between the two groups (*P* > 0.05).

**Table 2:** Baseline data of different C2h/C0 and insulin application.

Index	C_2h_/C_0_ < 2.5 ng/ml	C_2h_/C_0_ ≥ 2.5 ng/ml	*P*
Case number (*n*)	168	103	
Fasting blood glucose (mmol/L)	11.62 ± 3.24	8.71 ± 2.73	<0.001
Blood glucose at 2-h after meal (mmol/L)	21.03 ± 4.31	19.68 ± 4.10	0.01
BMI (kg/m^2^)	25.64 ± 3.38	24.74 ± 3.55	0.038
HbAlc (%)	11.36 ± 1.74	10.67 ± 1.88	0.003
TBa/h (U/h)	0.81 ± 0.14	0.74 ± 0.20	0.005
TDD/kg (U/kg)	0.54 ± 0.14	0.53 ± 0.14	0.734
%TBa (%)	0.50 ± 0.06	0.48 ± 0.05	0.02

*BMI* body mass index, *Tba* base amount in total, *TDD* total daily dose.

### The correlation analysis between the dosage of insulin and clinical indexes

As presented in Table 3, the Pearson correlation analysis revealed that there was a correlation between %TBa and BMI (*r* = 0.136, *P* < 0.05), and the difference was statistically significant. These results reveal that %TBa is positively correlated with BMI, but not with the other indexes. This also suggests that there was a significant correlation between C_2h_/C_0_ and waist circumference, HbAlc, FPG, and 2hPG (*r* = −0.137, −0.154, −0.471, and −0.172; *P* < 0.05 for all), and the difference was statistically significant. Furthermore, C_2h_/C_0_ was negatively correlated with waist circumference, HbAlc, FPG, and 2hPG, but not with the other indexes.

**Table 3:** Pearson correlation analysis of %TBa and C2h/C0 with each index.

Index	%TBa		C2h/C0	
	R	*P*	R	*P*
Age (years)	−0.064	0.293	0.025	0.68
Duration of disease (year)	−0.095	0.119	0.027	0.658
Waist circumference (cm)	0.073	0.229	−0.137	0.024
BMI (kg/m^2^)	0.136	0.025	−0.111	0.067
HbAlc (%)	−0.024	0.696	−0.154	0.011
FPG (mmol/l)	0.074	0.223	−0.471	<0.001
2hPG (mmol/l)	−0.057	0.353	−0.172	0.005

### Multivariate linear regression analysis

As presented in Table 4, multiple linear regression analysis was carried out with age, duration, BMI, HbA1c, FPG, and 2hPG as independent variables, and %TBa as the dependent variable. The results revealed that BMI and FPG were independent influencing factors of %TBa (β′ = 0.124 and 0.144, respectively; *P* < 0.05 for both). Furthermore, %TBa was positively correlated with BMI and FPG, but not with the other indexes. For the multiple linear regression analysis carried out with age, duration of T2DM, BMI, HbA1c, FPG, and 2hPG as independent variables, and C_2h_/C_0_ as the dependent variable, the results revealed that BMI and FPG were independent factors of C_2h_/C_0_ (β′ = −0.134 and −0.502, respectively; *P* < 0.05 for both). Furthermore, C_2h_/C_0_ was negatively correlated with BMI and FPG, but not with the other indexes, such as the age and duration of T2DM.

**Table 4:** Multivariate linear regression analysis.

	%TBa		C_2h_/C_0_	
Index	β′	*p*	β′	*p*
Age (years)	−0.006	0.934	0.021	0.726
Duration of disease (year)	−0.099	0.151	−0.012	0.846
BMI (kg/m^2^)	0.124	0.045	−0.134	0.014
HbAlc (%)	−0.034	0.608	−0.092	0.115
FPG (mmol/l)	0.144	0.042	−0.502	<0.001
2hPG (mmol/l)	−0.119	0.094	0.092	0.142

## Discussion

It has been suggested that short-term intensive therapy with CSII in T2DM can relieve glucotoxicity, protect the function of the remaining islet β-cells, and contribute to long-term blood glucose control, and is an important means of intensive insulin therapy^9^. However, few studies have been conducted on the initial dosage setting of pump insulin in T2DM, and most of these have mainly referred to the normal human insulin secretion mode and the application of patients with T1DM in foreign countries^8^. Previous studies^10–12^ have suggested that patients with good reserved β-cell function in the remaining islets would benefit more after intensive therapy. However, there is no simple index for the quantitative evaluation of the function of reserved islets. In clinical practice, C-peptide concentration is often used to evaluate the function of islet β-cells, which is not affected by exogenous insulin^13^. For patients with different functions of islet β-cells, the actual amount of insulin needed is quite different^14^. Therefore, it was speculated that the ratio of C_2h_/C_0_ might be used to evaluate the function of islet β-cells, and predict the insulin dosage.

The results of the present study revealed that the lower the C_2h_/C_0_ ratio, the worse the islet function, and the greater the needed basal insulin dosage. Furthermore, the higher the ratio of C_2h_/C_0_, the better the function of the islet, and the lower the needed dosage of basal insulin. In patients with T2DM, who received CSII therapy during hospitalization and had lower C_2h_/C_0_, the %TBa was nearly 50%, while in patients with normal C-peptide levels, the %TBa was approximately 40%. Ma Jing et al.^15^ reported that in 40% of patients recently diagnosed with T2DM, the %TBa was 40% when treated with CSII. At the same time, the study conducted by Zhang Xiuzhen^16^ revealed that the %TBa in Chinese patients with T2DM was 40–60% under intensive insulin pump therapy. Studies^17–19^ have revealed that most patients with T2DM still maintained a certain insulin secretion function, which could inhibit glycogen output from the liver. While in a small number of patients with T2DM in the early stage, due to the high glucose level, the islet function was inhibited, and insulin secretion was relatively reduced. Therefore, more basal amount was needed. Thus, according to the level of C-peptide, it may be necessary to increase the proportion of basal dosage for patients with poor islet function. The present study revealed that FPG and BMI were positively correlated with %TBa, but negatively correlated with C_2h_/C_0_. These were the independent factors before the initiation of insulin pump therapy. The higher the FPG in the patient at the time of admission, the more severe the impairment of β-cell function was, and the more insulin was needed. Meanwhile, patients with obesity have insulin resistance to a certain extent. The higher the BMI, the more basal insulin dosage was needed. Therefore, this suggests that the basal insulin dosage should be increased when setting for insulin pumps in obese patients.

There were some limitations in the present study. The continuous glucose monitoring system (CGMS) was not used before and after therapy. At the same time, the sample size of the present study was small. The results of the present study needs to be confirmed through further large sample, multicenter studies.

## Conclusion

The basal premeal dose ratio of T2DM with different C-peptide levels differs during intensive insulin pump therapy. Parameters that indicate the glycemic control and β-cell function should be taken into consideration for total insulin requirements.
